# Exploring sensory processing abilities in adults with acquired hearing loss

**DOI:** 10.1017/S0022215123001779

**Published:** 2024-05

**Authors:** Bilgehan Tekin Dal, Binnur Çetin, Eda Nur Şimşek, Gonca Bumin

**Affiliations:** 1 Department of Audiology, Faculty of Health Sciences, Gazi University, Ankara, Turkey; 2 Department of Occupational Therapy, Fizikon Medical Center, Konya, Turkey; 3 Department of Audiology, Kayseri Acıbadem Hospital, Kayseri, Turkey; 4 Department of Occupational Therapy, Faculty of Health Sciences, Hacettepe University, Ankara, Turkey

**Keywords:** Hearing loss, sensory processing, adult, sensorineural hearing loss

## Abstract

**Objectives:**

This study aimed to evaluate the sensory processing abilities of adults with acquired hearing loss and determine whether their sensory processing patterns differ from those of the general population and adults with normal hearing.

**Method:**

The study evaluated the sensory processing functions of 30 adults with acquired hearing loss using the Adolescent/Adult Sensory Profile and compared them with the sensory processing functions of 30 adults with normal hearing.

**Results:**

The results showed that individuals with hearing loss have a significantly higher sensitivity to stimuli related to motion, vision, activity and touch, exhibiting a low-registration sensory pattern and a sensation-avoiding pattern that differed from those of most individuals.

**Conclusion:**

Assessing sensory processing profiles can help identify specific sensory difficulties and inform individualised treatment plans. The study highlights the importance of considering sensory processing patterns in the management of hearing loss to improve overall well-being and quality of life for adults with hearing loss.

## Introduction

Hearing loss is a leading cause of disability worldwide, according to the Global Burden of Disease study.^1^ According to estimates from the World Health Organization, approximately 6.1 per cent of the world's population is affected by hearing loss, with the majority of those affected being adults.^2^

Hearing loss can be classified into three subcategories: conductive, sensorineural and mixed. These subcategories reflect different causes and mechanisms of hearing loss. Conductive hearing loss is caused by external and/or middle-ear impairments, while sensorineural hearing loss is caused by dysfunction in the cochlea or peripheral auditory nerve. Mixed hearing loss involves both conductive and sensorineural components.^3^ Sensorineural hearing loss is the most common type of hearing loss in adults and can be caused by various factors, such as aging, genetic mutations, noise exposure, ototoxic drugs, smoking and chronic conditions.^3,4^

Communication difficulties are the primary effect of hearing loss in adults and can lead to social isolation, reduced access to information and services, and emotional distress, ultimately impacting the quality of life of those affected.^3^

Studies have found a relationship between hearing loss and sensory processing in children, with up to 40 per cent of children with even mild or unilateral hearing loss experiencing sensory processing disorders.^5–8^ Sensory processing related to hearing is a crucial aspect of neurological development in children, enabling the formation of meaningful and purposeful behaviours, and successful participation in daily activities.^9,10^

In sensory processing disorder, individuals may demonstrate over- or under-responsivity to sensory stimuli in some or all sensory systems, including tactile, auditory, visual, olfactory, proprioceptive and vestibular systems. Two opposing hypotheses have been proposed to explain the effects of a deficit in one sensory system on other sensory systems.

The perceptual deficit hypothesis suggests that a deficit in one sensory system, such as the auditory system in the case of hearing loss, may affect the development and organisation of other sensory systems, resulting in poorer performance.^11^ For example, children with hearing loss have been found to have problems with vestibular function and lower scores in visual perception and praxis tasks.^5,6^

The second hypothesis is the sensory compensation hypothesis, which states that a deficit in one sensory system would lead to increased sensitivity in other sensory systems to compensate for the loss of input. According to the sensory compensation hypothesis, other sensory systems would show higher rates of responsiveness to sensory stimuli in auditory deficiency.^11^ For example, it has been documented that children with hearing loss have enhanced skin sensitivity and tactile discrimination abilities.^12^ Data about the relationship between hearing loss and sensory processing among adults are lacking in the literature.

This study aimed to investigate the sensory processing patterns of adults with hearing loss and compare them with healthy controls. One way to evaluate sensory processing is by analysing behavioural perspectives using sensory history, which is an assessment method that enquires about the rate of occurrence of behaviours and responses to sensory experiences in daily living situations. The advantages of sensory history over other evaluation methods include ease of application, evaluation of behaviour in natural environments and the ability for the person or family members to be active participants in the evaluations.^13^

In 1999, the sensory profile measurement method was developed to evaluate children's sensory processing skills between the ages of 3 and 10 years using sensory history.^14^ Based on Dunn's sensory processing model, the sensory profile was adapted to evaluate adults’ sensory processing skills in 2000. The model assumes that a person's sensory processing pattern depends on the relationship between their neurological thresholds and behavioural responses.

The neurological threshold refers to the amount of sensory input required for the nervous system to activate, and thresholds exist on a continuum with two poles: low and high. A low neurological threshold indicates that low-intensity stimuli are required for a person's neurons to fire and respond, while a high neurological threshold indicates that the person needs high-intensity stimuli or takes longer to react to the same stimulus.^9,15^ Behavioural responses are also on a continuum with two poles: active and passive. The behavioural response defines how people act in consideration of their thresholds.^14^ In the passive individual response, the person does not counteract sensory stimuli, while in the active individual response, the person acts to control the amount and type of sensory input.^15^

According to Dunn's model of sensory processing, the interaction of the neurological threshold continuum and the behavioural response continuum results in four sensory processing patterns: low-registration (reflecting behavioural passive responses to high neurological thresholds), sensation-seeking (reflecting active behavioural responses to high neurological thresholds), sensory-sensitivity (reflecting behavioural passive responses to low neurological thresholds) and sensation-avoiding (reflecting behavioural active responses to low neurological thresholds).^9^

People who have low-registration patterns fail to notice or have slow responses to sensations (as a result of having high neurological thresholds) and do nothing to capture additional sensations (as a result of using passive behavioural responses). For example, these people do not notice their faces or hands are dirty, and they also do not think to look at or touch their faces or hands to check for dirt.

People who have sensation-seeking patterns also do not notice stimuli as easily as people with low-registration patterns (as a result of having high neurological thresholds), but unlike those individuals, they enjoy rich sensory environments/activities (due to using active behavioural responses), which provide the level of stimulation needed to meet their own high thresholds. For example, these people may enjoy extreme sports or loud music, or engage in risky behaviour.

People with sensory sensitivity patterns are more sensitive to stimuli and detect sensory input that others may not notice (as a result of having high neurological thresholds). However, they do not actively seek to reduce or eliminate exposure to uncomfortable sensations (as a result of using passive behavioural responses). For example, these individuals may feel uncomfortable with what others consider normal levels of sounds, smells and movements, and they may be easily distracted by their sensory environment.

People who have sensation-avoiding patterns notice even small sensory inputs more easily than others (as a result of having low neurological thresholds) and tend to avoid exposure to stimuli as much as possible (as a result of using passive behavioural responses). For example, these people are easily startled by sudden movements or loud noises, and they often prefer to stay away from crowded places where others may move, talk or bump into them.^9,16^

Based on previous research on children, we hypothesise that adults with hearing loss will exhibit atypical sensory processing patterns. However, despite our literature review, we were unable to find any research that evaluates sensory processing abilities in adults with hearing loss. The investigation of sensory processing patterns in adults with hearing loss will provide valuable information to better understand the relationship between hearing loss and sensory processing, and to develop effective interventions to improve the quality of life of these adults.

## Materials and methods

This study was conducted with the approval of the Hacettepe university Non-Interventional Clinical Research Ethics Board (Ethical approval number: 2021/07-02). All participants provided written informed consent before completing self-report questionnaires and undergoing audiological evaluation.

### Participants

A total of 60 adults aged between 18 and 65 years were included in the study. Thirty individuals with acquired sensorineural hearing loss were recruited from the audiology outpatient unit in a hospital, while 30 individuals with normal hearing were selected as the control group. Individuals with acquired symmetric sensorineural hearing loss, a pure tone average (PTA) greater than 25 dB in the better ear and less than a 10 dB difference between air- and bone-conduction thresholds were included in the study group. In contrast, individuals with a PTA of 25 dB or less, better than 25 dB HL at all frequencies in both ears and less than a 10 dB difference between air- and bone-conduction thresholds were included in the control group. Exclusion criteria were the use of hearing aids, chronic tinnitus and/or dizziness, history of stroke, head injury, psychiatric illnesses, vision problems, lack of proficiency in speaking, reading and writing in their native language, and significant medical disorders and/or disabilities.

### Instruments

The study used the Hearing Handicap Inventory for Adults and the Adolescent/Adult Sensory Profile as assessment tools.

The Hearing Handicap Inventory for Adults is a 25-item self-assessment questionnaire that evaluates the emotional and social/situational aspects of perceived hearing disability. The scale consists of two subscales: the 13-item emotional subscale and the 12-item social/situational subscale. Each item is assigned a score of 0, 2 or 4 points for a ‘no’, ‘sometimes’ or ‘yes’ answer, respectively. The total score ranges from 0 to 100, with higher scores indicating a higher perception of hearing disability. The scale has been validated and reliability studies have been conducted, and a Turkish version is available.^17^

The Adolescent/Adult Sensory Profile is a 60-item self-assessment tool based on Dunn's sensory processing model. It evaluates behavioural patterns related to sensory processing and consists of six subsections: taste/smell processing, movement processing, visual processing, touch processing, auditory processing and activity level. Participants rate how often they react to sensory events on a five-point Likert scale (1 = almost never; 2 = sometimes; 3 = occasionally; 4 = often; 5 = almost always).

The Adolescent/Adult Sensory Profile questionnaire is divided into four subscales, which reflect different patterns of sensory processing. These quartiles include low-registration (e.g. ‘I don't smell things that other people say they smell’), sensory-sensitivity (e.g. ‘I feel uncomfortable wearing certain fabrics’), sensation-avoiding (e.g. ‘I only eat familiar foods’) and sensation-seeking (e.g. ‘I enjoy going to places with bright lights and colours’) sensory processing patterns. By analysing the responses, the Adolescent/Adult Sensory Profile can provide insights into an individual's sensory processing patterns and preferences. The evaluation is based on normative values created for three different age ranges: 11–18 years, 18–65 years and over 65 years old. Participants’ responses are classified as ‘much more than most people’, ‘more than most people’, ‘similar to most people’, ‘less than most people’ or ‘much less than most people’.^13,18^ Validity and reliability studies of the questionnaire have been conducted, and a Turkish version is available.^19^

### Data analyses

Descriptive analyses were performed using mean and standard deviation for numerical variables and frequency tables for ordinal and nominal variables. The Mann–Whitney *U* test was used to compare ordinal variables because the Adolescent/Adult Sensory Profile and Hearing Handicap Inventory for Adults scores were not normally distributed. The relationship between the quadrant sections of the sensory profile score (sensory sensitivity, sensation avoidance, sensory seeking and low registration) and age and PTA values was examined using Spearman correlation. A *p* value less than 0.05 was considered statistically significant. Normative values of Adolescent/Adult Sensory Profile scores were used to determine the percentage deviation from typical values.^16^ Statistical analyses were performed using SPSS software version 25.

## Results

The study group consisted of 30 adults (16 males and 14 females) with acquired sensorineural hearing loss, with an age range of 26–64 years (mean age, 52.10 ± 10.79 years). The control group comprised 30 adults (14 males and 16 females) with normal hearing thresholds, with an age range of 25–62 years (mean age, 49.00 ± 8.44 years). There were no significant differences in gender (*χ*^2^ = 0.267, *p* = 0.606) and age (*Z* = −1.510, *p* = 0.131) distribution between the two groups.

Pure tone average values calculated over 0.5, 1, 2 and 4 kHz ranged from 26.0 to 64.0 dB HL (mean, 38.17 ± 12.43 dB HL) in the right ear and 26.00 to 65.00 dB HL (mean, 38.70 ± 12.79 dB HL) in the left ear in the study group. Hearing loss degrees were determined based on PTA values: 53.3 per cent of the participants had mild hearing loss, 36.7 per cent had moderate hearing loss and 10.0 per cent had moderately severe hearing loss. The audiograms of all individuals with hearing loss were typical, with worsening hearing thresholds from mid frequencies (1 and 2 kHz) to high frequencies (4, 6 and 8 kHz). In the control group, the hearing thresholds of all participants were better than 25 dB HL at all frequencies, and their PTA values ranged from −1 to 23 dB HL in the right ear (mean, 8.57 ± 12.43 dB HL) and −3 to 24 dB HL in the left ear (mean, 8.03 ± 12.79 dB HL). The demographic characteristics and hearing threshold values of the study and control groups are provided in Table 1.

**Table 1.** Gender and age characteristics, and hearing thresholds (pure tone average) of the two groups

	Hearing loss (*n* = 30)	Normal hearing (*n* = 30)
Gender (*n* (%))		
– Male	16 (53.3)	16 (53.3)
– Female	14 (46.7)	14 (46.7)
Mean age ± SD (minimum–maximum) (years)	52.10 ± 10.79 (26–64)	49.00 ± 8.44 (25–62)
Pure tone average (dB)		
– Right, mean ± SD (minimum–maximum)	38.17 ± 12.43 (26.00-64.00)	8.57 ± 12.43 (−1.00–23.00)
– Left, mean ± SD (minimum–maximum)	38.70 ± 12.79 (26.00–64.00)	8.03 ± 12.79 (−3.00–24.00)

SD = standard deviation

In terms of the Hearing Handicap Inventory for Adults scores, participants with hearing loss had significantly higher total scores as well as higher scores in the emotional and social/situational subscales compared to the control group (*p* < 0.05). The total and subscale scores of the two groups and their comparison are presented in Table 2. A strong positive correlation was observed between PTA values and the total score of the Hearing Handicap Inventory for Adults (*r* = 0.817, *p* < 0.001), as well as the emotional (*r* = 0.835, *p* < 0.001) and social-situational subscales (*r* = 0.785, *p* < 0.001). This indicates that higher PTA values corresponded to a greater perception of hearing disability in emotional and social/situational areas.

**Table 2.** Hearing Handicap Inventory for Adults scores

	Hearing loss (*n* = 30)	Normal hearing (*n* = 30)	Mann–Whitney *U* test		
			*U*	*Z*	*p*
Total score					
– Mean ± SD	44.57 ± 22.49	6.67 ± 12.10	50.50	−5.977	<0.001
– Minimum–maximum	14–84	0–48			
Social/situational score					
– Mean ± SD	24.77 ± 10.17	3.40 ± 6.04	89.00	−5.432	<0.001
– Minimum–maximum	12–47	0–20			
Emotional score					
– Mean ± SD	24.77 ± 13.37	3.27 ± 6.49	28.50	−6.338	<0.001
– Minimum–maximum	2–46	0–28			

SD = standard deviation

The Adolescent/Adult Sensory Profile scores of the study and control groups are shown in Table 3. Individuals with hearing loss had significantly higher scores in the low-registration and sensory-sensitivity quadrants compared to those with normal hearing (*p* < 0.05). Spearman correlation analysis indicated a significant and moderate positive correlation between PTA values and low-registration scores. The scores for other quadrants did not show a significant correlation with age or PTA values (Table 4). In addition, there was a significant and moderate positive correlation between age and PTA values (*r* = 0.476, *p* < 0.001).

**Table 3.** Adolescent/Adult Sensory Profile scores

	Hearing loss (*n* = 30), mean ± SD	Normal hearing (*n* = 30), mean ± SD	Mann–Whitney *U* test		
			*U*	*Z*	*p*
Quadrant total raw score					
Low registration	46.43 ± 12.12	29.80 ± 6.73	116.000	−4.945	<0.001*
Sensation seeking	41.33 ± 7.84	44.33 ± 6.95	337.000	−1.674	0.094
Sensory sensitivity	43.03 ± 7.69	38.83 ± 6.87	303.500	−2.170	0.030*
Sensation avoiding	41.50 ± 7.21	40.60 ± 9.12	381.000	−1.022	0.307
Sensory modality score					
Taste/smell	22.27 ± 1.98	21.00 ± 2.74	325.000	−1.866	0.062
Movement	24.30 ± 4.82	21.10 ± 3.86	287.000	−2.420	0.016*
Visual	28.17 ± 4.65	26.10 ± 4.65	310.000	−2.079	0.038*
Touch	39.87 ± 7.16	31.03 ± 7.76	167.500	−4.184	<0.001*
Activity	29.40 ± 5.58	25.97 ± 3.82	213.500	−3.508	<0.001*
Auditory	28.30 ± 4.41	28.37 ± 5.81	419.500	−0.460	0.645

SD = standard deviation. *p < 0.05

**Table 4.** Relationships between age, pure tone average values and sensory processing scores

	Age		Pure tone average	
	*r*	*p*	*r*	*p*
Quadrant total raw score				
Low registration	0.175	0.181	0.585	<0.001*
Sensation seeking	0.209	0.109	−0.163	0.212
Sensory sensitivity	−0.101	0.441	0.147	0.261
Sensation avoiding	0.047	0.722	0.157	0.232

*r* = correlation co-efficient. *p < 0.05

When compared with normative data, 83 per cent of individuals with hearing loss had scores that were different from those of most individuals in terms of low registration, while 17 per cent had scores similar to those of most individuals. For sensation avoiding, 63 per cent of individuals with hearing loss had scores that were different from those of most individuals, and 37 per cent had scores similar to those of most individuals (Table 5).

**Table 5.** Distribution of scores for each quadrant of the two groups of participants

	Much less than most people (*n* (%))		Less than most people (*n* (%))		Similar to most people (*n* (%))		More than most people (*n* (%))		Much more than most people (*n* (%))	
Quadrant	HL	NH	HL	NH	HL	NH	HL	NH	HL	NH
Low registration	0 (0)	4 (13)	1 (3)	2 (6)	5 (16)	21 (70)	7 (23)	3 (10)	56 (17)	0 (0)
Sensation seeking	10 (30)	5 (16)	5 (16)	6 (20)	15 (50)	19 (63)	0 (0)	0 (0)	0 (0)	0 (0)
Sensory sensitivity	0 (0)	1 (3)	1 (3)	2 (6)	15 (50)	16 (53)	6 (20)	11 (36)	8 (26)	0 (n)
Sensation avoiding	0 (0)	0 (0)	1 (3)	0 (0)	11 (36)	18 (60)	15 (50)	5 (16)	3 (10)	7 (23)

HL = hearing loss; NH = normal hearing

Overall, the results indicate that individuals with hearing loss perceive greater hearing disability in emotional and social/situational areas, and have significantly different sensory processing patterns compared to those with normal hearing.

## Discussion

This study aimed to investigate the sensory processing patterns of adults with hearing loss and compare them to those without hearing loss. Four key findings were reported. Firstly, it was found that individuals with hearing loss have a greater tendency toward a low-registration sensory pattern. Secondly, they were found to have a significantly higher sensitivity to stimuli related to movement, vision, activity and touch. Thirdly, when comparing adults with hearing loss to normative data from the Adolescent/Adult Sensory Profile manual, 83 per cent of the study participants exhibited a low-registration pattern that differed from that of most individuals. Lastly, it was found that as hearing sensitivity decreased, there was an increase in the perception of hearing difficulties in the emotional and social/situational areas.

Importantly, to the best of our knowledge, no prior studies have investigated sensory processing patterns in adults with hearing loss. The findings of this study provide valuable information that can be used to develop more effective interventions and support for adults with hearing loss.

Our findings revealed that individuals with hearing loss scored higher in the low-registration and sensory-sensitivity quadrants compared to those without hearing loss, as determined by quadrant analysis. These results are consistent with previous studies demonstrating differences in sensory processing in children with hearing loss.^7,19^ Alkhamra and Abu-Dahab found that children with hearing loss exhibited greater sensory processing difficulties.^7^ Similarly, Schum reported that individuals with hearing loss show greater sensory processing difficulties, which may contribute to communication challenges and reduced quality of life.^19^ Based on the information presented, we infer that the findings from this study provide new insights into the sensory processing difficulties experienced by adults with hearing loss. While previous studies have primarily focused on children with hearing loss, our study underscores that sensory processing difficulties can also manifest in adults with hearing loss. These results, in conjunction with previous research, suggest that sensory processing difficulties play a significant role in addressing communication difficulties and reduced quality of life in adults with hearing loss. Further research is needed to better comprehend the impact of sensory processing difficulties on adults with hearing loss and to develop targeted interventions to address these challenges.

While our study found that adults with hearing impairment scored higher in certain quadrants related to sensory processing, only low-registration scores were significantly positively correlated with PTA values. Individuals who score high on low registration may have difficulty reacting quickly to stimuli, especially less salient or weak stimuli. Consequently, they may miss important cues, leading to challenges in appropriately responding to environmental stimuli. For instance, they may fail to notice changes in their surroundings, experience difficulty hearing certain sounds, struggle to distinguish flavours and may not detect bothersome odours.^9^ When we evaluate our findings in light of this information, individuals with greater hearing loss may be more likely to exhibit a low-registration pattern, which may in turn contribute to further difficulties in perceiving auditory stimuli.

It is crucial to interpret these results within the context of hearing impairment and consider the potential interplay between sensory processing patterns and hearing loss. It is important to note the fact that individuals with hearing loss have difficulty perceiving auditory stimuli or distinguishing between different sounds as a result of their hearing loss. Nonetheless, sensory processing patterns, such as low registration, can interact with hearing loss and further contribute to these difficulties. A comprehensive approach involving assessment and intervention strategies addressing both hearing loss and sensory processing difficulties may therefore be necessary to provide tailored support for individuals with hearing loss.

Ultimately, further research is needed to determine the exact relationship between hearing loss, low-registration scores and challenges in perceiving auditory stimuli. Understanding this relationship can aid in the development of interventions to enhance the sensory processing skills of individuals with hearing loss. Nevertheless, our findings shed light on the sensory processing abilities of adults with acquired hearing loss and suggest a potential connection between hearing loss and sensory processing. Further research in this area could contribute to the development of effective interventions to enhance sensory processing skills in individuals with hearing loss.

In conclusion, our results suggest that individuals with greater hearing loss may be more likely to exhibit a low-registration pattern, which can lead to difficulties in perceiving auditory stimuli, particularly when those sounds are relatively quiet. It is important to consider that individuals may exhibit different sensory patterns for different types of sensory stimuli,^20^ therefore it may not be feasible to classify individuals into specific sensory patterns. However, gaining a better understanding of the unique sensory patterns of populations with specific health conditions can be valuable for developing targeted and individualised intervention programmes.^21^ By identifying the specific sensory processing patterns of populations with specific health conditions, such as hearing loss, healthcare professionals and audiologists can develop individualised treatment plans that address their specific needs and preferences. This may include interventions that target specific sensory modalities, such as auditory training or sensory-based strategies, as well as interventions that focus on enhancing overall communication abilities and quality of life. It is important to note that while this study provides valuable information, it may not be feasible to classify individuals into specific sensory patterns because of the variability of sensory processing among individuals, therefore gaining a better understanding of the unique sensory patterns of populations with specific health conditions is essential for developing effective intervention programmes.

Our study also investigated the relationship between hearing loss and sensory modalities. We found that individuals with hearing loss may experience hypersensitivity in movement, activity, and visual and tactile processing. This indicates that individuals with hearing loss may experience heightened sensitivity to stimuli in these areas, which can significantly impact their daily lives. Specifically, we found that adults with hearing loss showed sensory hypersensitivity in the processing of movement, which suggests that vestibular functions may also be affected. This is consistent with previous studies showing abnormalities in vestibular and balance functions in individuals with hearing loss.^8^

Our study also found sensory hypersensitivity in activity processing in adults with hearing loss, which is consistent with previous research in children with hearing loss. This finding suggests the potential influence of psychosocial factors on sensory processing. Studies have shown that anxiety and depression are common among individuals with hearing loss and may cause differences in sensory processing through attentional biases and emotional regulation.^22–26^

However, it is important to note that our study only found an association between hearing loss and sensory processing profiles, rather than a causal relationship. It is possible that problems in one sensory system may make an individual rely more on other sensory systems and become more sensitive to their inputs, rather than causing sensory processing difficulties. Alternative explanations for our findings, such as compensatory mechanisms, should therefore be considered.

Future studies should investigate the relationship between the type and intensity of activities, and their psychological effects on individuals with hearing loss. This may provide a more nuanced understanding of how hearing loss impacts sensory processing and psychosocial functioning in daily life, and inform the development of personalised interventions that address specific types and intensities of activities and their psychological effects.

The Adolescent/Adult Sensory Profile is a tool used to assess sensory processing in daily life. In our study, we used this tool to evaluate sensory processing profiles in adults with hearing loss. According to the Adolescent/Adult Sensory Profile user manual,^16^ we interpreted the scores and found that adults with hearing loss had differences in the low-registration and sensation-avoiding quadrants. This suggests that adults with hearing loss may experience sensory processing difficulties or disorders, similar to children with hearing loss.^7,8,27^

In our study, we found that adults with hearing loss score higher than most people on the sensation-avoiding quadrant of the Adolescent/Adult Sensory Profile. Individuals who exhibit higher scores in the sensation-avoiding quadrant demonstrate a pattern of sensory processing behaviour characterised by actively avoiding or being sensitive to certain types of sensory stimuli. For instance, these individuals may avoid specific foods or textures, prefer loose clothing to evade feelings of constraint or avoid noisy crowds. This finding is important as it sheds light on how individuals with hearing loss perceive and process sensory information in their daily lives.

Adults with hearing loss encounter challenges in processing auditory information, particularly in congested and noisy environments, which can lead to feelings of stress and anxiety. Consequently, they may be more inclined to engage in sensation-avoiding behaviours as a means of managing these emotional responses. This behavioural strategy can serve as a method for individuals to distance themselves from the adverse effects of hearing loss or as a way to establish a sense of control.

Wearing hearing aids may offer a potential solution to address sensory avoidance behaviour among these individuals. Nonetheless, a pattern of sensation avoiding among those with hearing loss could potentially compound the difficulties already stemming from their hearing impairment. For instance, some adults with hearing loss might opt to forego wearing hearing aids as a result of a pre-existing pattern of sensation avoiding, or they may persist in such avoidance even while using properly fitted hearing aids.

A similar concept applies to the low-registration pattern. In our study, we noted that adults with hearing loss exhibited higher scores in the low-registration quadrant compared to most people in the general population, and their scores also exceeded those of the age-matched control group. The low-registration pattern observed in adults with hearing loss may exacerbate their challenges in perceiving auditory stimuli as a result of their hearing impairment. Consequently, this pattern could persist even when wearing well-fitted hearing aids.

Because of these considerations, a comprehensive approach involving assessment and intervention strategies addressing both hearing loss and sensory processing difficulties is likely necessary to provide specialised support for individuals with hearing impairment.

In this study, our primary focus was to explore sensory processing patterns among adults with hearing loss. Given this specific objective, we did not include individuals who use hearing aids in our research. Our aim was to investigate the impact of acquired hearing loss in isolation, without the potential confounding influence of hearing-aid usage.

However, it is important to acknowledge that well-fitted hearing aids can indeed enhance auditory perception and alleviate challenges in noisy environments, potentially affecting sensory processing patterns independently of hearing loss. Therefore, in future research, an exploration of the sensory processing profiles of adults with hearing loss who wear hearing aids, while accounting for factors such as hearing-aid type and duration of use, would yield valuable insights. Notably, to our knowledge, our study is among the first to examine sensory profiles in adults with acquired hearing loss, and our findings suggest that sensory processing difficulties may commonly manifest among this population.

It is imperative to underscore that the interpretation of Adolescent/Adult Sensory Profile scores ideally should be conducted on an individual basis, as stipulated in the Adolescent/Adult Sensory Profile user manual. Additionally, it is worth noting that while the sensory profile has been extensively employed across various populations, including children with hearing loss, it has not been specifically validated for use in adults with sensorineural hearing loss. Despite this limitation, our findings offer preliminary evidence suggesting that the Adolescent/Adult Sensory Profile could serve as a valuable tool for assessing sensory processing in individuals with hearing loss.

The observed disparities in sensory processing patterns between individuals with and without hearing loss substantiate the notion that sensory processing challenges may be prevalent among adults with acquired hearing loss. Nevertheless, further investigation is warranted to corroborate the findings of this study and to ascertain the utility of the Adolescent/Adult Sensory Profile in this particular population.

Our study found that individuals with hearing impairment experience more difficulties in emotional and social situations than individuals with normal hearing, as demonstrated by significantly higher scores on the Hearing Handicap Inventory total score and the social/situational and emotional subscale scores. Similar findings have been reported in studies investigating the translated versions of Hearing Handicap Inventory for Adults, such as the Malay version by Zam Zam *et al*.^28^ and the Brazilian version by Aiello *et al*.^29^ Although these studies differ from ours in terms of the scope of the investigation, they also reported significant differences in total and subscale scores between individuals with hearing impairment and those with normal hearing, which is consistent with our findings.

Moreover, we found a strong and significant positive correlation between the PTA values and the Hearing Handicap Inventory for Adults total score and social/situational and emotional subscale scores, indicating that as the PTA values increase, there is an increase in the perception of hearing impairment in emotional and social/situational domains. Our findings are consistent with previous research, including the study by Ferrari *et al*. that found that hearing impairment was associated with increased social isolation, decreased social functioning and increased emotional distress.^30^ The degree of hearing impairment was also found to be a significant predictor of social and emotional functioning, with greater hearing loss associated with greater social and emotional difficulties.

- This study sheds light on the sensory processing difficulties experienced by adults with hearing loss, specifically in low register and sensory sensitivity
- There is a negative impact of hearing impairment on emotional and social functioning, with greater hearing loss associated with greater social and emotional difficulties
- The importance of personalised interventions that address specific needs and preferences is emphasised for improving overall well-being in individuals with hearing impairment
- The Adolescent/Adult Sensory Profile score is a valuable tool for assessing sensory processing in daily life, but its interpretation should be done on an individual basis
- The importance of providing appropriate support and interventions to improve the quality of life and overall well-being of individuals with hearing impairment is highlighted

It is crucial to provide appropriate support and interventions for individuals with hearing impairment, taking into account the negative impact of acquired hearing loss and the degree of hearing loss on emotional and social well-being. By addressing these challenges, we can improve the quality of life and overall well-being of adults with hearing loss and enhance their social and emotional functioning.

## Conclusion

This study found that adults with hearing loss may have different sensory processing patterns compared with adults with normal hearing. Specifically, adults with hearing loss may be more likely to have a low-registration pattern and to experience hypersensitivity in movement, activity, and visual and tactile processing. While our study underscores the prevalence of these patterns, it is essential to note that the interplay between sensory processing and hearing loss is complex and multifaceted. Furthermore, our investigation into the emotional and social implications of hearing impairment underscores the need for targeted strategies to enhance the overall well-being and quality of life of individuals with hearing loss.

These findings provide invaluable insights that can guide future research endeavours and the development of interventions that optimise sensory processing skills and promote holistic well-being for this population. Healthcare professionals and audiologists can develop individualised treatment plans that address the specific needs and preferences of individuals with hearing loss. As the understanding of sensory processing patterns in adults with hearing loss advances, these insights hold the potential to drive innovative approaches that enhance communication, engagement and emotional resilience in their daily lives.
